# Imaging of a Case of Extramedullary Solitary Plasmacytoma of the Trachea

**DOI:** 10.1155/2011/687203

**Published:** 2011-09-22

**Authors:** M. Garelli, C. Righini, C. Faure, A. Jankowski, C. Brambilla, G. R. Ferretti

**Affiliations:** ^1^Clinique Universitaire de Radiologie et Imagerie Médicale, CHU de Grenoble, 38043 Grenoble Cedex, France; ^2^Université Joseph Fourier, 38041 Grenoble Cedex 9, France; ^3^INSERM U 823, Institut A Bonniot, La Tronche, France; ^4^Clinique Universitaire d'ORL, CHU de Grenoble, 38700 La Tronche, France; ^5^Département de Pathologie, CHU de Grenoble, 38700 La Tronche, France; ^6^Clinique Universitaire de Pneumologie, Grenoble, France

## Abstract

We describe a case of extramedullary tracheal plasmacytoma that was incidentally discovered in a 73-year-old man on a PET scan performed for assessing the extent of colon cancer. CT scan showed the tumor; multiplanar reformation coupled with virtual bronchoscopy allowed proper treatment planning. The tracheal tumor was resected during rigid bronchoscopy. Relevant investigations excluded multiple myeloma. Follow-up CT showed persistent thickening of the tracheal wall, but there has been no recurrence after one-year followup.

## 1. Introduction

Extramedullary plasmacytoma (EMP) is a rare plasma cell malignancy described in soft tissues outside of the bone marrow. It arises in different sites in the body especially in the upper airway [1]. The most common sites are the nose and paranasal sinuses. Primary laryngeal and tracheal lesions are very rare, and only a few cases of tracheal EMP have been described [1–4]. We report a case of solitary tracheal plasmacytoma incidentally discovered on a PET scan.

## 2. Case Report

**Figure 1:**
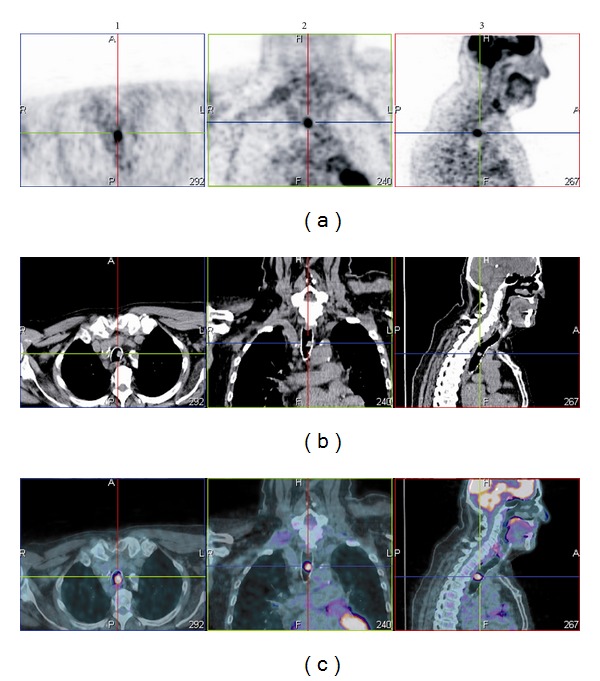
PET scan shows a localized FDG uptake in the middle third of the trachea. (a): PET, (b): CT, (c): PET-CT. (1): axial view, (2): coronal, (3): sagittal.

This 73-year-old nonsmoker man had a history of colon cancer, for which he underwent a surgical treatment during autumn 2009. On a follow-up abdominal CT, a possible liver metastasis was discovered for which a PET scan was required which showed a localized and unique FDG uptake within the middle third of the trachea (Figure 1). He complained of a dry cough but denied any other respiratory complaints. Physical examination was unremarkable.

**Figure 2:**
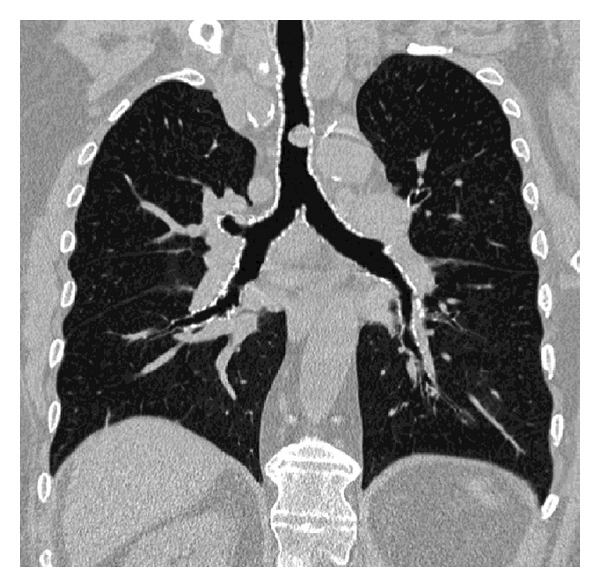
CT scan. CT coronal reformation shows the longitudinal extent of the tumor, its location 35 mm above the carina, and the severity of tracheal narrowing (40%).

**Figure 3:**
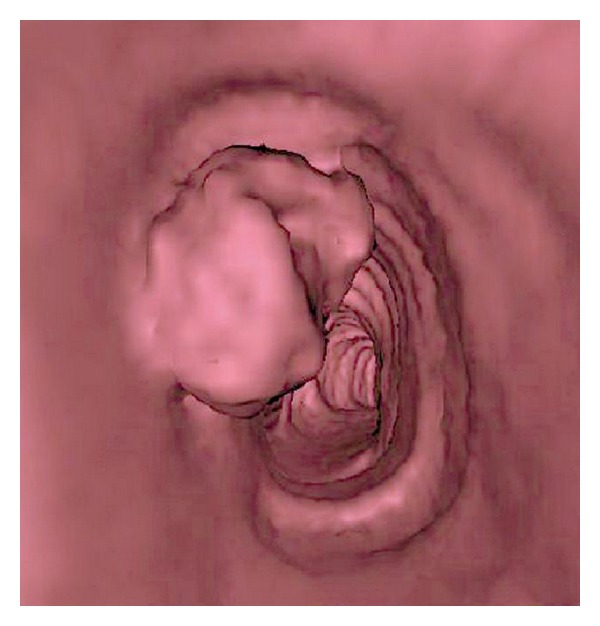
CT scan. Virtual endoscopy demonstrates an endoluminal view of the tracheal tumor.

The patient underwent a CT scan of the chest with an acquisition performed at the end of inspiration. A mass, 15-mm in diameter, arising from the left anterior wall of the middle third of the trachea was confirmed. This exophytic tumor was well limited and grew in contact with the tracheal cartilage, without evidence of cartilage invasion. Coronal reformation showed the longitudinal extent of the plasmacytoma, location of its upper part was 8.5 cm distal to vocal cords, and its distal part was 3.5 cm above the carina (Figure 2). Virtual bronchoscopy demonstrated the severity of the airway narrowing (Figure 3). No lymph node enlargement was observed within the mediastinum.

Fiber optic bronchoscopy revealed a fleshy tracheal mass occluding approximately 40% of the tracheal lumen and located 10 cm distal to the vocal cords. Pathological examination of the biopsies suggested an extramedullary plasmacytoma. On protein electrophoresis there was a diffuse elevation in the gamma region, but the immunoglobulin levels were normal on immunoelectrophoresis, excepting a discrete elevation of IgG and IgM levels, without monoclonal band. Urine contained no Bence-Jones protein. Bone marrow biopsy showed an excess of plasma cells (4%), without dysmorphic cells. No lytic bone lesion was observed on plain radiography which eliminates multiple myeloma. 

**Figure 4:**
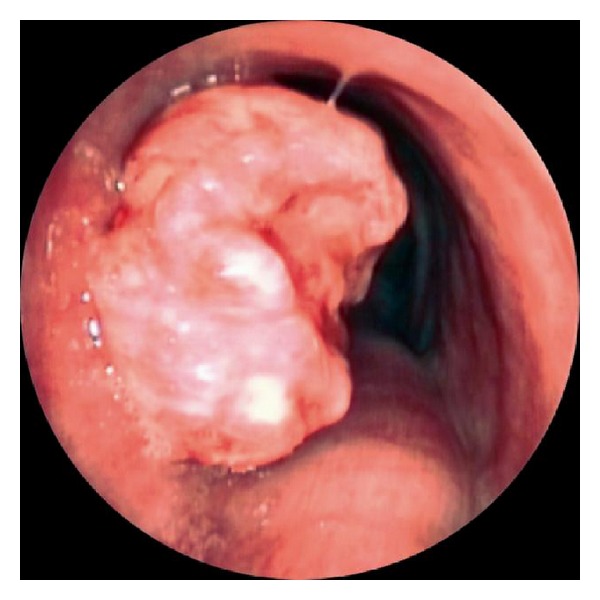
Snapshot by tracheoscopy during the surgery shows an obstructive and fleshy tracheal mass.

**Figure 5:**
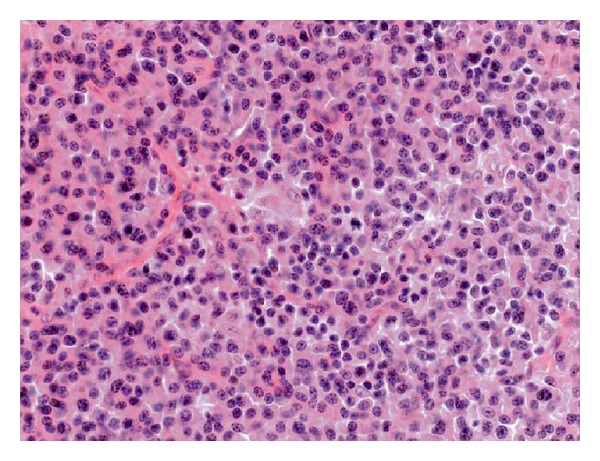
Photomicrograph of surgical specimen. Diffuse infiltrate of neoplastic monoclonal well-differentiated plasma cells is present associated with many deposits of amyloid in the stroma (HES ×200).

**Figure 6:**
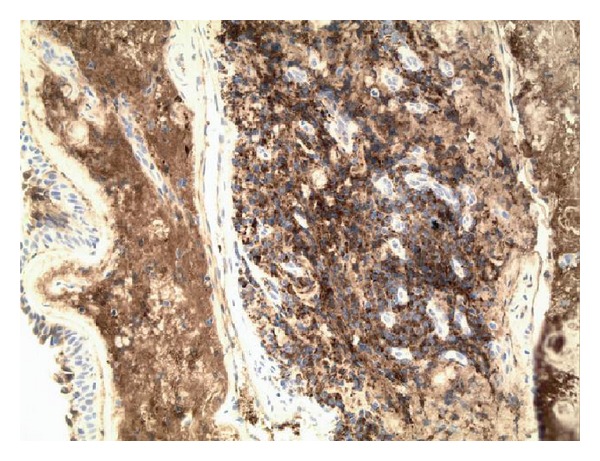
Photomicrograph of surgical specimen. The plasma cells express cytoplasmic immunoglobulin with light chain restriction. They also express CD138, marker characteristically positive for plasma cells (immunohistochemistry, kappa ×200).

The patient was treated endoscopically under general anesthesia using a rigid ventilating bronchoscope (Karl Storz, Tuttlingen, Germany). The endoscopic intraoperative view showed an obstructive tracheal mass with a small insertion pedicle (Figure 4). The mass was excised with the extremity of the bronchoscope, and the base of insertion was coagulated with a rigid monopolar probe. The final histological examination showed a diffuse infiltrate of neoplastic monoclonal well-differentiated plasma cells (Figure 5). At immunohistochemistry the plasma cells express cytoplasmic immunoglobulin with light chain restriction and CD138, marker characteristically positive for plasma cells (Figure 6). It confirmed a well to moderately differentiated plasmacytoma. Postoperatively, repeat CT scan showed smooth thickening of left lateral wall of trachea. An endoscopy was performed and revealed an irregular and inflammatory area in place of the fleshy lesion. Histology of this area was negative, and the patient remains well, with no evidence of recurrence 12 months after treatment.

## 3. Discussion

Tracheal tumors are uncommon and represent around 1 to 2% of all respiratory tract tumors [5]. Squamous cell carcinoma and adenoid cystic carcinoma are the most frequent malignant tracheal tumors [4, 5]. Extramedullary plasmacytoma belongs to plasma cell tumors and represents around 3% of them [4]. It usually occurs in 50-to-60-year-old patients and affects predominantly men (sex ratio male/female of 3 : 1 to 5 : 1) [6, 7]. Common symptoms of tracheal tumors are related to airway obstruction. Clinically, the dyspnea is becoming evident when the narrowing of the airway is over 75% in diameter [4]. Other signs include coughing, voice changing, haemoptysis, stridor, acute respiratory failure, and expiratory wheezing [5, 7]. A case of tracheal plasmacytoma has been mistaken for asthma [3] and a case of pharyngeal plasmacytoma for sleep apnea syndrome [7].

On reported EMP cases, 80% of EMP are located in the upper aerodigestive tract and appear as a soft tissue mass [4, 8]. They usually involve the submucosal lymphoid tissue of nasopharynx or paranasal sinuses [7–9]. Rare cases have been described in the larynx [10], hypopharynx, cervical glands, trachea, oesophagus, cervical lymph nodes, middle ear, and mastoid. Even more rarely, they are described in the trachea [1, 2]. 

The diagnostic approach of those tracheal tumors was completely modified by the introduction of CT scan as CT allows evaluation of the extent within the lumen, airway wall, and mediastinal structures [5]. Two-dimensional postprocessing, that is, multiplanar reformations in sagittal, coronal, or oblique planes, are useful for assessing the type, degree, and longitudinal extent of the narrowing of the airway as well as the location of the tumor, the distance from the cricoid cartilage and to the carina. Virtual bronchoscopy shows an endoluminal view of the tumor with an excellent correlation with conventional bronchoscopy [5]. In this case the stenosis-to-lumen ratio was estimated at around 40% by both techniques. Because of the lack of distinguishing clinical and radiological features, the final diagnosis is done by pathologic examination, which demonstrated that the tumor is composed of sheets of neoplastic monoclonal plasma cells expressing cytoplasmic immunoglobulin with light chain restriction at immunohistochemistry and CD138 [6, 8]. Negative results of a postoperative myeloma survey and negative results of testing a bone marrow biopsy are essential for ruling out multiple myeloma [6, 8, 10]. There must be less than 5% plasma cells in bone marrow biopsy.

Treatment of tracheal plasmacytomas remains controversial as radiotherapy [4, 10] or surgery alone, and surgery followed by radiotherapy [2] are current options. In our case, the patient had an endoscopic resection. The surgery can be performed via low tracheostomy under direct vision in cases of major obstruction [4] or secondary in cases of incomplete excision. Some authors [9] stressed the fact that surgery can achieve a satisfactory local treatment, but that radical excision is often difficult, which was not the case in our patient. The novelty comes here from the monitoring of the patient: although a persistent thickening of tracheal wall was described following endoscopic excision and local coagulation, there was no histological relapse at 12 month after treatment. Adjuvant chemotherapy does not seem to have a role in the local treatment but can be used in case of relapse or dissemination [8–10].

Even if the prognosis of EMP seems to be better than that of disseminated myeloma, patients require careful monitoring and long-term followup as a local recurrence or a progression to multiple myeloma has been described in up to 20% of cases [7]. The 5-year survival rates are between 30 and 82% in EMP [9].

This is, to our knowledge, the first reported case of tracheal extramedullary plasmacytoma discovered incidentally on a PET scan. The case demonstrates the value of CT scan for preoperative planning of endoscopic surgery. Annual followup is mandatory, not to mistake an evolution to multiple myeloma or a local recurrence.
